# Obstetric Thromboprophylaxis: The Swedish Guidelines

**DOI:** 10.1155/2011/157483

**Published:** 2011-11-22

**Authors:** Pelle G. Lindqvist, Margareta Hellgren

**Affiliations:** ^1^Department of Obstetrics and Gynecology, Karolinska University Hospital, 14186 Stockholm, Sweden; ^2^Department of Obstetrics and Gynecology, Sahlgrenska University Hospital Gothenburg, 41345 Gothenburg, Sweden; ^3^Department of Antenatal Care, Primary Care South Bohuslän, 43102 Molndal, Sweden

## Abstract

Obstetric thromboprophylaxis is difficult. Since 10 years Swedish obstetricians have used a combined risk estimation model and recommendations concerning to whom, at what dose, when, and for how long thromboprophylaxis is to be administrated based on a weighted risk score. In this paper we describe the background and validation of the Swedish guidelines for obstetric thromboprophylaxis in women with moderate-high risk of VTE, that is, at similar or higher risk as the antepartum risk among women with history of thrombosis. The risk score is based on major risk factors (i.e., 5-fold increased risk of thromboembolism). We present data on the efficacy of the model, the cost-effectiveness, and the lifestyle advice that is given. We believe that the Swedish guidelines for obstetric thromboprophylaxis aid clinicians in providing women at increased risk of VTE with effective and appropriate thromboprophylaxis, thus avoiding both over- and under-treatment.

## 1. Introduction

The incidence of obstetric venous thromboembolism (VTE) in the Nordic countries is estimated at 10 to 13 cases per 10000 pregnancies, half of them diagnosed during the first six weeks after birth [1, 2]. VTE is one of the most common causes of maternal death [3, 4] and leads to morbidity in the form of postthrombotic syndrome in up to 50–60% [5, 6]. Several factors are known to increase the risk of obstetric VTE, such as personal or family history of VTE, thrombophilia, older age, high body mass index (BMI), immobilization, surgery, smoking, nulliparity, and cancer [1, 2]. 

Thromboprophylaxis during pregnancy usually consists of daily subcutaneous injections of low molecular weight heparin (LMWH), in combination with compression stockings [7–10]. There are several recommendations concerning how to identify women at high risk of VTE during pregnancy and the puerperium. Some of them divide women into low-medium and high-risk groups [7, 8] and others are based on weighted risk scores [9, 10]. Risk assessment and management of obstetric thromboprophylaxis differ in different countries.

The aim of this paper is to describe the background and validation of the Swedish guidelines for obstetric thromboprophylaxis in women with moderate-high risk of VTE. A weighted risk score for estimation of obstetric VTE risk has been used in Sweden for around 10 years. Recommendations concerning to whom, at what dose, when, and for how long thromboprophylaxis is to be administered are based on this risk score. A small number of special cases (women with antithrombin deficiency, antiphospholipid syndrome (APS) with prior VTE or prior multiple VTE, as well as those on chronic warfarin treatment) cannot be managed according to the risk score. These women are estimated to be at very high, that is, 15% or higher, risk of obstetric VTE. They are recommended “two-dose” thromboprophylaxis, which is not included in this paper. Thromboprophylaxis in relation to legal abortion, miscarriage, or ectopic pregnancy is also excluded from this paper.

## 2. Methods

The available literature from 1996 to 2000 on risk factors for obstetric VTE was reviewed. Two different risk models were constructed, one a weighted risk score based on major risk factors associated with a five-fold increased risk or a multiple thereof [11, 12], and the other an individualized computerized risk assessment using and including estimates of the absolute risk [11]. Hem-ARG, the Swedish Association of Obstetricians and Gynecologists (SFOG) reference and working group on hemostatic disturbances in obstetrics and gynecology, chosed the weighted risk score model. Swedish guidelines based on this model were published in 2004 [12], and established as a National Guideline by SFOG in 2009. The guidelines were revised in 2010-2011, resulting in the version presented here. 

### 2.1. Risk Assessment of Obstetric VTE

To facilitate the assessment of VTE risk during pregnancy and the puerperium, a scoring system was created by adding the weight of various risk factors (Table 1) for obstetric VTE [1]. An approximately five-fold increased risk of VTE during pregnancy, shown by the odds ratio (OR) for a number of risk factors for VTE, yields one risk score point (Table 2). Three conditions, cesarean section (CS), preeclampsia, and abruptio placentae, are only considered to be risk factors during the puerperium. Since all risk factors cannot be included in an algorithm, the variable “other major risk factor” is included (Table 2) and may be used, according to the clinician's decision, in cases of malignancy, extreme obesity, and so forth. The woman's risk score is calculated as the sum of the points. Women with risk score 1 are at a five-fold increased risk, risk score 2 (two variables with 1 point or one variable with 2 points) are at a 25-fold increased risk and risk score 3 entails a 125-fold increased risk, and so forth, compared to risk score 0, which means no increased risk (Table 2). Women with a previous VTE or APS without VTE are given 4 risk points, regardless of other risk factors. The total risk score is the basis for determining when, how, and at what dose thromboprophylaxis should be administered (Table 3). 

During pregnancy it is mainly women with prior VTE that reach the risk score level for thromboprophylaxis. A heterozygous carrier of Factor V Leiden (FVL) without any other risk factors has 1 risk point and would not be recommended ante- or postpartum prophylaxis. However, if she also has first-degree heredity for VTE, one more risk point is added, resulting in a risk score of 2, leading to a recommended one week of postpartum thromboprophylaxis. If she also undergoes CS, she is given 3 risk points (FVL, heredity, and CS), resulting in a risk score of 3, and she would be recommended six weeks of postpartum thromboprophylaxis. 

The model also takes into account the variation in risk during pregnancy and the puerperium, that is, about a five-fold higher risk postpartum, at its highest during the first week after delivery [2, 13]. This could make different durations of postpartum thromboprophylaxis possible, for example, one week (risk score 2), six weeks (risk score 3 or 4), or three months “very high risk” (Table 3). Some weak but significant risk factors are not considered in this model, such as smoking, blood group other than O, multiple gestation, varicose veins, parity, and acute CS (as compared to elective CS). Several of these risk factors have been taken into account in another model [11] (Table 1).

## 3. Recommendations

As is evident from Table 2, LMWH thromboprophylaxis is recommended at a risk level corresponding to the antenatal risk of women with one prior VTE. This risk level can also be attained by the simultaneous existence of several risk factors in a woman with no history of VTE. The differences in ante- and postpartum risk (postpartum risk is about five-fold higher) and the quickly decreasing risk after the first postpartum week are taken into account in the scoring system. In the following, we will present “normal-dose” thromboprophylaxis, as recommended for women at moderate-high risk of VTE during pregnancy. 

LMWH thromboprophylaxis is recommended as soon as pregnancy is confirmed in women with previous VTE or a risk score of at least 4 points. If thromboprophylaxis is only indicated postpartum, it is initiated about four hours after uncomplicated childbirth and no laboratory testing is required. In case of bleeding complications, an individual assessment is recommended. During pregnancy and after vaginal delivery, LMWH is usually injected in the abdominal wall. Pharmacological treatment is always combined with compression stockings, grade 1 knee socks that are used as early as possible during pregnancy and continued at least 12 weeks postpartum. In the case of postthrombotic syndrome, grade 2 stockings are recommended. 

### 3.1. “Normal Dose” Thromboprophylaxis during Pregnancy

Before the initiation of thromboprophylaxis, APTT, PT (INR), and platelet count are checked. Platelet count is repeated after two weeks of thromboprophylaxis to rule out heparin-induced thrombocytopenia (HIT). LMWH thromboprophylaxis is administered at the “normal dose” (Table 5) once daily throughout pregnancy without monitoring anticoagulant effect to women with a VTE risk score of at least 4 and body weight ≤90 kg (in early pregnancy). A higher initiation dose is recommended (Table 5) for women with a body weight exceeding 90 kg; they should also test anti-FXa activity three hours postinjection, about 2 weeks after the initiation or dose change. An anti-FXa activity between 0.20 and 0.45 U/mL is the goal. If necessary, the dose is adjusted, reduced, or increased by half the “normal dose.” If the initial anti-FXa activity is appropriate, no further verification is required, in the absence of abnormal weight gain or obstetric complications. The recommendations of different doses according to weight are based on pharmacokinetic studies and clinical experience of recurrences [14, 15].

In addition to the usual visits to the midwife, women with prior VTE are usually scheduled for two extra visits to an obstetrician: one at the beginning of pregnancy for planning and initiation of thromboprophylaxis and one at about 34 weeks of gestation for planning of thromboprophylaxis in relation to delivery; lifestyle information is also given (see below).

Short-term thromboprophylaxis is recommended during situations entailing temporary risk increases (e.g., fractures with casts, strict bed rest). Women with ovarian hyperstimulation syndrome (OHSS) after in vitro fertilization are given “normal-dose” thromboprophylaxis during hospitalization and for six weeks after leaving hospital.

### 3.2. “Normal Dose” Thromboprophylaxis during Delivery and Postpartum

On arrival at the maternity ward, APTT, PT (INR), and platelet count are assessed and the time of the last LMWH injection is recorded. Spontaneous vaginal delivery is usually planned. LMWH is discontinued during active labor and the next injection is administered about four hours after birth. Thromboprophylaxis is initiated about four hours after CS in cases with risk score >2. The first week after CS, the injection is given in the thigh in order to avoid hematomas in the abdominal wall.

After delivery, the same dose of LMWH is given as during pregnancy and the thromboprophylaxis is continued for one (risk score 2) or six weeks (risk score 3 or 4), depending on the risk score. After complicated delivery, individualized initiation of thromboprophylaxis is mandatory. Cessation of bleeding must always take precedence before thromboprophylaxis, but it is important to start thromboprophylaxis again as soon as possible after the condition is stabilized. LMWH thromboprophylaxis is usually recommended postpartum but sometimes the women want to change to warfarin in order to avoid injections. Warfarin thromboprophylaxis makes it even more important to be vigilant for bleeding complications postpartum if abnormal bleeding occurs. The effect of warfarin may vary considerably; higher doses and more monitoring are necessary in the immediate postpartum period, compared to non-pregnant conditions [16]. If warfarin is being considered for women with protein C or S deficiency, it is initiated at a lower dose than normal and LMWH administration should continue simultaneously for at least one week.

## 4. Discussion

### 4.1. Thromboprophylaxis at Different Risk Levels

The risk score is a clinical aid for avoiding over- and under-treatment. The proportion of women with risk score 2 in a pregnant population is 0.9% during pregnancy and 4.0% postpartum (Table 4) [17]. The reason for this discrepancy is that CS, preeclampsia, and abruptio placentae only raise the risk of VTE postpartum (Table 2). If the risk score is the basis for clinical management, less than 5% of all women will receive LMWH thromboprophylaxis, that is, less than 0.5% of the population will receive ante- and postpartum thromboprophylaxis and 4–4.5 % will only be given postpartum thromboprophylaxis.

### 4.2. Efficacy of LMWH Thromboprophylaxis

Without thromboprophylaxis, VTE recurs during pregnancy in about 10% (5% during pregnancy and 5% postpartum) among women with a prior VTE [18]. Thromboprophylaxis according to our guidelines prevents 88% of VTE in women with one previous episode [18]. However, these women are still at a six-fold increased risk of both ante- and postpartum VTE [18]. The highest risk is during the post-treatment period, 43–100 days after delivery and termination of thromboprophylaxis [18]. During the postpartum period, there are many women without VTE history at the same risk as those with prior VTE during the antepartum period. Theoretically, the risk score should be able to reduce the number of postpartum pulmonary embolisms by two thirds [19]. Since two thirds of lethal pulmonary embolisms occur postpartum and 1/6 of maternal mortality is caused by pulmonary embolism, the risk of maternal mortality may theoretically be lowered by 1/12 if the algorithm is followed [19].

Increased maternal age is an established risk factor for VTE. A five-fold increased risk is more associated with maternal age >40 than with the commonly quoted age of >35 [1]. Regarding thrombophilias, we adapted the algorithm according to practice in Sweden. Protein C and protein S deficiencies were given 2 points each. Almost all women with thrombophilia, but without history of VTE, were tested because of family history of VTE (defined as first-degree relative(s) with onset before age 60), yielding a risk score 2 + 1 = 3. They would usually be recommended at least six weeks of postpartum thromboprophylaxis. Homozygous FVL was given 3 risk points and these women would always be recommended at least six weeks of postpartum thromboprophylaxis.

## 5. Cost-Effectiveness

Following the guidelines has been shown to be cost-effective, especially when it comes to postpartum thromboprophylaxis for those with two and three risk points, for whom the cost of thromboprophylaxis is between 25% and 50% of the cost of thrombotic complications [19]. It is important to remember that the cost-effectiveness data is only valid if the guidelines are followed strictly. 

### 5.1. Lifestyle Advice

Fertile women with history of VTE have an approximately 1–3% annual risk of recurrence [18, 20]. All women with prior VTE should be given lifestyle advice. Regular exercise cuts the recurrence risk by half [21]. Exercise may be recommended in the form of walking briskly for around 30 minutes a day, in addition to normal everyday physical activity. Swimming and water aerobics are suitable right up until birth during uncomplicated pregnancy. The risk of VTE is 50% higher during the winter months than the rest of the year [22]. This may be an effect of vitamin D deficiency in a large proportion of the population, especially during winter [22]. Women with habitual sun exposure are at 30% lower risk of VTE as compared to those avoiding sun exposure [22]. Smoking increases the risk of VTE and smokers should be advised to quit [1, 23]. Women with BMI <25 are at a 30% lower risk of VTE than overweight (BMI 25–30) women and at three-fold lower risk than obese (BMI 30+) women [1, 23]. By maintaining normal weight, preferably through exercise, the risk of blood clots is kept low [1].

### 5.2. Amendments

In the revised (2011) version of the recommendations, inflammatory bowel disease was included as a risk factor [24, 25] and abruptio placentae was added as a risk factor postpartum [2, 26]. Presently, profuse blood loss and placenta previa are being assessed as potential major risk factors. In the prior version of the guidelines, women with a provoked VTE after a temporary situation, such as orthopedic surgery, initiated their prophylaxis at 20 weeks gestation. However, after several recurrences before 20 weeks, we considered that the difference in risk between women suffering a VTE after a temporary and a permanent risk factor was not sufficient to lead to differing clinical management. All women with prior VTE are recommended thromboprophylaxis from early pregnancy, which also makes the guidelines easier to follow. 

We conclude that the Swedish guidelines for obstetric thromboprophylaxis aid clinicians in providing women at increased risk of VTE with effective and appropriate thromboprophylaxis, thus avoiding both over-and under-treatment.

## Figures and Tables

**Table 1:** Risk factors and their odds ratios (ORs) för VTE (1,2).

	Prevalence (%)	Pregnancy OR	Postpartum OR
Overweight (BMI > 28)^1^			
>28 BMI	12.8	5	5
Familial thrombosis (first-degree relatives)			
yes	5.1	5	5
Age			
<20	2.5	1.0	2.5
≥20–<35	85.2	1.0	1
≥35	12.4	1.0	1.2
Smoking			
yes	21.0	1.2	1.2
Parity			
Primapara	41.3	2.3	1.1
1 birth	35.4	1	1
2 births	15.8	1.3	1.7
>2 births	7.3	2.6	1.8
Preeclampsia			
Yes	2.0		3–5
Cesarean section			
Yes	11.0		4.9
FV Leiden			
Noncarrier	89.1	1	1
Heterozygotes	10.6	5	5
Homozygotes	0.3	25–100	25–100
Protein S or protein C deficiency	0.1	5–25	5–25
Prothrombin gene mutation	2.0	5	5
Hyperhomocysteinemia		2–5	2–5

^1^BMI > mean + 1SD in early pregnancy is regarded as overweight.

**Table 2:** Risk points, each corresponding to a five-fold increased risk, are added, yielding a risk score.

1 point	2 points	3 points	>4 points^5^	Very high risk^6^
Heterozygote FV Leiden	Prot S deficiency	Homo FV Leiden	Prior VTE	Mechanical heart prosthesis
Heterozygote FII mut	Prot C deficiency	Homo FII mut	APS without VTE^7^	Chronic warfarin prophylaxis
Overweight^1^	Immobilization^4^			Antithrombin deficiency
Cesarean Section				Recurrent VTE
Heredity for VTE^2^				APS with VTE^7^
Age >40 years				
Preeclampsia				
Hyperhomocysteinemia^3^				
Abruptio placenta				
Inflammatory bowel disease				
Other major riskfactor				

Homo: Homozygote, mut: mutation, VTE: venous thrombembolism.

APS: Antiphospholipidsyndrome, Prot: protein, FV: faktor V, FII: factor II (prothrombin).

^1^Overweight = (BMI >28 in early pregnancy).

^2^VTE in first-degree relative <60 years of age.

^3^Homocysteine >8 *μ*mol/L in pregnancy.

^4^During cast treatment for fracture or strict bed rest short-term thromboprophylaxis is recommended.

^5^Women with prior VTE or APS without VTE have risk score 4 independent of other risk factors.

^6^Women in this group are classified as “very high risk” and are not scored.

^7^Women with APS are recommended low dose (75 mg) acetylsalicylic acid in addition to LMWH.

The risk score is formed by adding each point to a score between 0 and maximum 4 (for >4 points).

**Table 3:** Management based on risk score (the sum of riskpoints in Table 1).

Risk score	
0	No thromboprophylaxis
1	No thromboprophylaxis
2	Short-term LMWH thromboprohylaxis after delivery (7 days)* or during immobilization
3	6 weeks of LMWH thromboprophylaxis after delivery*
≥4	Antepartum thromboprophylaxis, and at least 6 weeks postpartum**
“Very high risk”	High-dose antepartum prophylaxis and at least 12 weeks of postpartum prophylaxis***

*Initiated 4 hours after delivery.

**Women with history of VTE initiate thromboprophylaxis in early pregnancy.

***Thromboprophylaxis is initiated as early as possible and sometimes before pregnancy. Only women with antithrombin deficiency, chronic warfarin prophylaxis, recurrent VTE, antiphospholipidsyndrome with VTE, and those with mechanical heart prosthesis are included in this group.

**Table 4:** Distribution of risk score* in a pregnant population, at delivery [17], among women with postpartum VTE, or postpartum pulmonary embolism [19].

Riskscore*	Reference group during		Reference group at		Post partum VTE		Post partum pulmonary	
	pregnancy		delivery		group		embolism	
	(*n* = 2384)		(*n* = 2384)		(*n* = 37)		(*n* = 11)	
	*n*	%	*n*	%	*n*	%	*n*	%
0	1940	81.4	1758	74.0	10	27.0	2	18.2
1	414	17.4	515	22.0	9	24.3	1	9.1
2	22	0.9	96	4.0	10	27.0	4	36.4
3	0	0	7	0.3	3	8.1	2	18.2
≥4	8	0.3	8	0.3	5	13.5	1	9.1

*Risk score based on anamnestic variables.

**Table 5:** LMWH dose.

	Body weight* (Kg)	Dalteparin s.c. U/24 h	Tinzaparin s.c. U/24 h	Enoxaparin mg/24 h
Risk score = 2 to 4***				
“Normal-dose” thromboprophylaxis	<90	5000	4500	40
	>90	7500**	75 U/kg**	60**

“Very high risk” of VTE****				
	<50	2500 × 2**		20 × 2**
“High-dose” thromboprophylaxis	50–90	5000 × 2**	175 U/kg**	40 × 2**
	>90	7500 × 2**		60 × 2**

*Initial maternal weight at the antenatal care unit.

**Initial doses.

***Maximum score is 4.

****measurable anti-FXa activity during the whole day, that is, >0.1 U/mL plasma before next dose.
